# Acetyltransferase p300 regulates atrial fibroblast senescence and age‐related atrial fibrosis through p53/Smad3 axis

**DOI:** 10.1111/acel.13743

**Published:** 2022-12-05

**Authors:** Xiao‐Yan Gao, Ying‐Yu Lai, Xue‐Shan Luo, De‐Wei Peng, Qiao‐Qiao Li, Hui‐Shan Zhou, Yu‐Mei Xue, Hui‐Ming Guo, Jun‐Fei Zhao, Hui Yang, Su‐Juan Kuang, Zhao‐Yu Wang, Meng‐Zhen Zhang, Chun‐Yu Deng, Shu‐Lin Wu, Fang Rao

**Affiliations:** ^1^ Guangdong Cardiovascular Institute, Guangdong Provincial People's Hospital Guangdong Academy of Medical Sciences Guangzhou China; ^2^ Provincial Key Laboratory of Clinical Pharmacology, Research Center of Medical Sciences, Guangdong Provincial People's Hospital Guangdong Academy of Medical Sciences Guangzhou China; ^3^ Department of Pharmacy The People's Hospital of Hezhou Hezhou China

**Keywords:** atrial fibrillation, fibrosis, p300, p53, senescence, Smad3

## Abstract

Atrial fibrosis induced by aging is one of the main causes of atrial fibrillation (AF), but the potential molecular mechanism is not clear. Acetyltransferase p300 participates in the cellular senescence and fibrosis, which might be involved in the age‐related atrial fibrosis. Four microarray datasets generated from atrial tissue of AF patients and sinus rhythm (SR) controls were analyzed to find the possible relationship of p300 (EP300) with senescence and fibrosis. And then, biochemical assays and in vivo electrophysiological examination were performed on older AF patients, aging mice, and senescent atrial fibroblasts. The results showed that (1) the left atrial tissues of older AF patients, aging mouse, and senescence human atrial fibroblasts had more severe atrial fibrosis and higher protein expression levels of p300, p53/acetylated p53 (ac‐p53)/p21, Smad3/p‐Smads, and fibrosis‐related factors. (2) p300 inhibitor curcumin and p300 knockdown treated aging mouse and senescence human atrial fibroblasts reduced the senescence ratio of atrial fibroblasts, ameliorated the atrial fibrosis, and decreased the AF inducibility. In contrast, over‐expression of p300 can lead to the senescence of atrial fibroblasts and atrial fibrosis. (3) p53 knockdown decreased the expression of aging and fibrosis‐related proteins. (4) Co‐immunoprecipitation and immunofluorescence showed that p53 forms a complex with smad3 and directly regulates the expression of smad3 in atrial fibroblasts. Our findings suggest that the mechanism of atrial fibrosis induced by aging is, at least, partially dependent on the regulation of p300, which provides new sights into the AF treatment, especially for the elderly.

## Graphical abstract

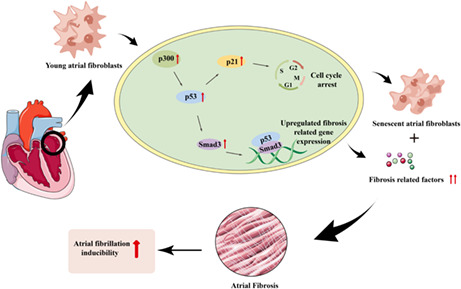

**NOVELTY AND SIGNIFICANCE**

**What is known?**

- Atrial fibrosis and the incidence of atrial fibrillation increase with age.
- The transcript coactivator and acetyltransferase p300 is involved in cellular signaling pathways to control cell senescence and fibrosis.
- Tumor suppressor, p53, can activate p21 to induce cell senescence and can also interact with Smad3 to promote the expression of fibrosis cytokines.

**What new information does this article contribute?**

- Atrial tissue p300 levels increase in aged mice along with the atrial fibrosis and high incidence of atrial fibrillation.
- p300 participates in the age‐related fibrosis through regulating p53/Smad3 pathway.
- Pharmacological inhibition or gene knockdown of this p300‐dependent pathway attenuates the onset and progression of age‐induced atrial fibrillation.

## 1 INTRODUCTION

Atrial fibrillation (AF) is the most common cardiac arrhythmia worldwide. Its prevalence increases rapidly with age. The incidence of AF in adults was estimated 1% before age of 50 and reached 10–20% over age 80 in the world (Rahman et al., 2014). It is generally known that atrial fibrosis can lead to abnormal atrial conduction, and reentry pathway thus contribute to the development and maintenance of AF (Andrade et al., 2014; Dzeshka et al., 2015). Progressive fibrosis is a hallmark of aging in various organs including the cardiovascular system (Calabresi et al., 2007; Gagliano et al., 2007; Meschiari et al., 2017). Progressive age‐related deposition of collagen in the heart of human and animal models was reported (Gazoti Debessa et al., 2001; Thomas et al., 2001). Senescent cardiac fibroblasts are the main senescent cells in cardiac fibrosis (Meyer et al., 2016). Aging‐related cardiac fibrosis is mainly due to the imbalance of cardiac collagen synthesis and degradation, as well as enhanced collagen cross‐linking (Thomas et al., 2001). However, the underlying molecular mechanisms of age‐related atrial fibrosis are still unclear.

The transcript coactivator and acetyltransferase p300 is a chromatin‐modifying enzyme that modulates the expression and function of genes involved in cell cycle regulation, apoptosis, growth, and development (Ghosh & Varga, 2007; Goodman & Smolik, 2000). In the cardiovascular system, elevated levels of p300 are associated with cardiac hypertrophy, cardiomyopathy, fibrosis, heart failure, and pathologies of cardiac aging (Bugyei‐Twum et al., 2014; Rai et al., 2019; Sunagawa et al., 2011; Wei et al., 2008). Even though some studies indicated that p300 plays a pivotal role in accelerated cardiac aging including myocardial infarction, diabetic cardiomyopathy, and myocardial matrix remodeling (Feng et al., 2016; Miyamoto et al., 2006; Sunagawa et al., 2011), the role of p300 in the natural aging heart, especially the atrium, is still unclear. In addition, p300 is an essential regulator of profibrogenic signal in pathological fibrogenesis. It was demonstrated that p300 was involved in TGF‐β signal pathway which lead to the skin fibrogenesis in scleroderma patients and p300 interacted with phospho‐Smad2/3 in human glomerulonesphritis (Ghosh et al., 2000; Kassimatis et al., 2010). In the cardiovascular system, p300 could promote Smad2 acetylation, activate TGF‐β, and lead to myocardial fibrosis and hypertrophy in diabetic cardiomyopathy rats (Bugyei‐Twum et al., 2014). However, it is uncertain whether p300 also plays an important role in aging‐related cardiac fibrosis, especially atrial fibrosis.

As a multifunctional tumor suppressor, p53 can activate p21 and induce cell senescence, which could be regulated by p300 (Zhang et al., 2015). p300 can also interact with Smad3 to promote the transcription of fibrosis‐related factors. The p53‐Smad3 transcription axis is established as a novel treatment target in renal fibrosis (Overstreet et al., 2014; Samarakoon et al., 2012). In the myocardial infarction model of aged mice, cardiac fibroblasts were obviously clustered around the infarcted myocardium and characterized by the significant up‐regulation of p53 protein (Zhu et al., 2013). These findings suggest that p300 and p53 are not only critical in cell senescence but also important in fibrosis. In the present study, we hypothesized that p300 would activate p53 through p53/p21pathway and p53/Smad3 complex to mediate the cardiac fibroblast senescence and aging‐related fibrosis.

In this study, we used bioinformatic methods to analyze original genetic data from the atrial tissue of AF patients and sinus rhythm (SR) controls and found out that EP300/TP53 might be potential mechanism for senescence and fibrosis in AF. Then, the protein expression of p300/p53/Smad3 and fibrosis factors in human atrial appendages, aging animal, and senescence atrial fibroblasts model were detected. Moreover, loss‐of‐function and gain‐of‐function models were used to determine an essential role of p300 and its potential signaling pathways in atrial fibroblast senescence and aging‐related fibrosis. Our results suggest that p300 plays a pivotal role in atrial fibroblast senescence and aging‐related fibrosis through p53/p21 and p53/Smad3 pathway.

## 2 METHODS

### 2.1 Datasets

Four microarray datasets, GSE115574, GSE79768, GSE14975, and GSE41177 were analyzed. In the GSE79768 dataset, the expression matrix of a total of 13 cases was acquired from the atrial tissue of patients, including 7 patients with AF, and 6 with SR. GSE115574 involved 28 cases of samples from patients with AF and 31 cases of control. The GSE41177 dataset contained 16 patients with AF and 3 SR patients. GSE14975 involved 5 atrial tissue samples from AF patients and 5 from SR patients. The series matrix file and data table of the microarray platforms GPL570 were also downloaded.

### 2.2 Data preprocessing and differentially expressed genes (DEGs) identification

The microarray data preprocessing, containing background correction and normalization, was performed by using the “Affy” package in R (Gautier et al., 2004). Afterwards, “sva” package of the R software was applied to batch normalization and merge four datasets (Leek et al., 2012). Then, “ggplot2” and “Heatmap” package were used to determine the expression differences of EP300 and its related molecules between the AF and SR (Gu et al., 2016).

### 2.3 Construction of EP300's co‐expression network and conduction of correlation analysis between EP300, senescence, and fibrosis makers

To further examine the biologic mechanism of EP300 in AF, EP300 co‐expression network was constructed by using the search tool of STRING database with the minimum required interaction score equal to medium confidence (0.40), and active interaction sources were set to default. The resulting network was illustrated using Cytoscape (v3.7.2) with degree‐sorted circular layout (Gu et al., 2014). To identify the most significant modules and top 10 neighbors with EP300, MCODE and CytoHubba plugin were applied. The top 10 high‐degree proteins and its interaction degree were visualized by bar plot. Furthermore, we have downloaded the cellular senescence and regulation of extracellular matrix organization GO term gene sets from the MSigDB “c5.bp.v7.0.symbols.gmt,” including 67 cellular senescence‐related and 35 fibrosis‐associated genes. Then the expression matrixes of corresponding genes in AF group was extracted from GEO datasets for further analysis. Firstly, we performed the correlation analysis between EP300 and two phenotype‐related genes former description (102 genes). Secondly, a matrix correlation analysis between phenotype‐related genes using the Pearson method was performed. Lastly, correlation analysis between EP300, senescence, and fibrosis markers was applied by “Circlize” packages.

### 2.4 An analysis of ROC curves and nomograms

In order to investigate the potential clinical significance of EP300 as well as its associated genes, we used “pROC” packages to determine the diagnostic values of each of them (Robin et al., 2011). Moreover, in order to improve the capacity in distinguishing AF patients from all samples, we constructed the combined diagnosis model of four genes (EP300, TP53, COL1A1, and COL3A1). A multivariate regression formula was built based on the four genes' expression value and their regression coefficients under the merged datasets. Finally, a nomogram was constructed based on the selected predictive factors by using the “rms” package in R to predict the prevalence of AF (Iasonos et al., 2008).

### 2.5 Function enrichment analysis

In order to explore the biological function of EP300 and its co‐expression genes, Gene Ontology (GO) and Kyoto Encyclopedia of Genes and Genomes (KEGG) pathway analyses were performed by using R “GO plot.” The results were visualized with chord graphs. Enrichment significance thresholds were set at an adjusted *p*‐value below 0.05. Gene set enrichment analysis (GSEA) was performed using clusterProfiler (R package) and visualized by “gseaplot” package (Yu et al., 2012). The median of EP300 expression level as a cut‐off value, the 61 AF samples in merged datasets were divided into low and high expression groups. The potential biological function of EP300 (high vs. low) was identified using GSEA to determine whether defined lists (or sets) of genes exhibited a statistically significant bias in their distribution within a ranked gene list. An |NES| > 1 and FDR <0.25 were deemed as statistically significant. (NES, normalized enrichment score; FDR, false discovery rate).

### 2.6 Patients

The studies were conformed to the Helsinki declaration and were approved by the ethics committee of the Guangdong Provincial People's Hospital, Guangdong Academy of Medical Sciences (No. GDREC20160128H). All subjects were given written informed consent. Patients with pneumonia or other infectious diseases were excluded. Atrial appendages (AAs) were obtained from individuals undergoing cardiopulmonary bypass surgery or thoracoscopic surgery in Guangdong Provincial People's Hospital (Guangzhou, Guangdong, China). Specimens from 16 patients with chronic AF (≥6 months) [young AF group (18–49 years old, *n* = 8), old AF group (≥50 years old, *n* = 8)] and a control group of 16 patients with SR [younger SR group (18–49 years old, *n* = 8), older SR group (≥50 years old, *n* = 8)] were used in this study. The baseline characteristics of included individuals are shown in Table 1. The AAs were fixed in 4% formalin for immunohistochemistry, or frozen in liquid nitrogen and stored at −80°C for Western blot analysis or cell culture.

**TABLE 1:** Baseline characteristics of the included individuals

	SR young	SR old	AF young	AF old
Number	8	8	8	8
Age (y)	30.88 ± 1.78	60.50 ± 2.00**	36.25 ± 1.58	57.75 ± 2.47**, ^$$^
Male (*n*)	6	3	2	3
LAD (mm)	43.25 ± 2.35	42.50 ± 2.98	55.63 ± 2.78**, ^##^	50.75 ± 1.33*, ^#^
LVEF (%)	60.00 ± 5.96	58.38 ± 3.20	60.75 ± 2.24	60.13 ± 2.89
Hypertension (*n*)	0	2	0	0
Diabetes (*n*)	0	0	0	0
CAD (*n*)	0	3	0	0
MVR (*n*)	7	4	8	7
AVR (*n*)	4	3	4	5
CABG (*n*)	0	2	0	0
β‐blocker (*n*)	6	7	2	5
Digitalis (*n*)	8	6	7	5
ACEI/ARB (*n*)	4	4	2	4
Diuretics (*n*)	8	8	8	8

*Note*: Data are mean ± SEM.

Abbreviations: ACEI/ARB, angiotensin‐converting enzyme inhibitor/angiotensin receptor antagonist; AF, atrial fibrillation; AVR, aortic valve replacement; CABG, coronary artery bypass graft; CAD, coronary artery disease; LAD, left atrial diameter; LVEF, left ventricular fraction; MVR, mitral valve replacement; SR, sinus rhythm.

* *p* < 0.05

** *p* < 0.01 vs. SR young;

^#^ *p* < 0.05

^##^ *p* < 0.01 vs. SR old;

^$$^ *p* < 0.01 vs. AF young.

### 2.7 Animals

The study was approved by the Research Ethics committee of Guangdong Provincial People's Hospital, Guangdong Academy of Medical Sciences (No. GDREC2016128A). All animals received care in compliance with the Guide for the Care and Use of Laboratory Animals published by the US National Institutes of Health (NIH publication no. 85‐23, revised 1996). Male C57BL/6 mice were purchased from the animal research center of Guangzhou University of Chinese Medicine and housed in a temperature and humidity‐controlled room on a 12 h light/dark cycle, with ad libitum access to food and water. Mice were raised to 18–20 months old to establish the aging model. At 12‐month‐old, mice were randomly received either oral curcumin non‐fat milk powder pill (50 or 100 mg/kg/day curcumin, Cayman, Canada) or an equivalent weight of non‐fat milk powder pill for 6 months. Mice heterozygous for a targeted p300 allele (p300−/+) were generated by crossing p300 floxed mice with CAG‐cre mice (B6‐CAG‐Cre, a mouse model of systemic expression of CRE enzyme driven by CAG promoter; Jiangsu GemPharmatech Co., Ltd, China). Floxed, but Cre‐negative, littermates were used as experimental controls (wild type, WT). The cardiac structure and function were evaluated by echocardiography under isoflurane anesthesia.

### 2.8 Electrophysiological study protocol

Mice were anesthetized by intraperitoneal administration of 0.03–0.04 mg/g pentobarbital and fixed on a temperature‐regulated heat pad (RWD Life Science Inc, Shenzhen, China) to maintain the body temperature at 37.0 ± 1.0 °C. Surface and intracardiac ECG were displayed and recorded by the iWorx Data acquisition and analysis system (iWorx Systems, Dover NH, USA) for detailed analysis and measurement. The jugular vein was exposed, and a 1.2‐French octopolar catheter (4 electrode with 1 mm circular electrodes; electrode distance, 0.5 mm) was advanced through the vein into the right atrium (RA). The atrial pacing threshold is determined by applying 1 ms pulse width and a pacing rate 10 beats/min faster than normal. The S1‐S2 interval was progressively reduced by 2‐ms in each pacing train. For AF inducibility, a 15 s atrial burst pacing (2‐fold diastolic capture threshold, 20 ms basic cycle length (BCL), and 1 ms pulse width) was performed and repeated 10 times. Only AF lasting for at least 1 s was considered as successful AF induction. AF duration was measured from initiation of AF to that of the first sinus beat. AF inducibility was calculated as the percentage of successful AF induction. After completion of electrophysiology study, animals were sacrificed by cervical dislocation under anesthesia, and the hearts were collected.

### 2.9 Isolation of human atrial fibroblast, transfection, gene silencing, and overexpression

Human atrial fibroblasts were isolated from left atrial appendage (LAAs) of SR patients. Briefly, the tissue specimens were fully shredded and attached in the flasks for 90–120 min, cultured under complete media with 4.5 g/L glucose and glutamine (Lonza), 25 mM HEPES (Sigma Aldrich, pH 7.4), 100 μg/mL streptomycin, 100 U/mL penicillin and 10% FBS (Linaris, LaborChemie, Vienna, Austria) at 37°C in 5% CO_2_‐95% air. Cells were cultured for up to passage11 (P11) to establish the senescence cell model. Plasmid and siRNA specific for human p300, p53, or scrambled control (Shanghai Genechem Co. Ltd, China) were transfected into atrial fibroblasts using lipofection to knock down or over‐express the protein expression.

### 2.10 Western blotting and co‐immunoprecipitation (Co‐IP)

Atrial tissue or atrial fibroblasts were homogenized in RIPA lysis buffer (Beyotime Biotechnology, Shanghai, China) and centrifuged at 12,000 rpm for 15 min at 4°C. The proteins (30 μg) were fractionated on 8% or 10% SDS–polyacrylamide gels and transferred to PVDF membranes (Millipore, Merck, Germany) and blocked with dried skimmed milk powder in Tris‐buffered saline Tween (TBST) for 1 h at room temperature. The membranes were incubated with primary antibody (Please see details in Supplementary materials) and horseradish peroxidase‐conjugated secondary antibody, and the proteins were then detected using the ECL chemiluminescence system (Merck Millipore, Darmstadt, Germany).

For Co‐IP, the crude cell lysate was centrifuged at 3500 rpm for 15 min at 4°C. To preclear the lysate, 1 ml of supernatant was exposed to 30 μl of protein A/G agarose beads (Upstate, Billerica, MA, USA) for 30 min at 4°C. Fifteen percent of total protein was taken out to detect the expression of input protein. Immunoprecipitations were carried out in a volume of 1 ml for 400 μg of total protein. A total of 8 μg of rabbit anti‐Smad3 was added to the precleared lysates, and the samples were incubated for 1 h, rocking gently at 4°C. A total of 50 μl of protein A/G agarose beads were added, and the samples were incubated on a rocker for 1 h at 4°C. The antibody–protein A/G agarose complex was spun down at 3500 rpm for 5 min and washed with lysis buffer three times. After the final spin, 100 μl of lysis buffer and 25 μl of 4 × Laemmli buffer were added. Co‐immunoprecipitation was tested by Western blot analysis using a rabbit p53 antibody. As negative controls, additional reactions were carried out in parallel with an equivalent amount of rabbit IgG bound to the protein A/G agarose beads.

### 2.11 SA‐β‐gal staining

The Senescence β‐Galactosidase Staining Kit (Cell Signaling Technology, Danvers, MA; cat no: #9860) was used to detect β‐galactosidase activity in cultured cardiac fibroblasts and heart tissues at pH 6.0, according to the kit instructions.

### 2.12 Masson trichrome staining and immunofluorescence

For analysis of collagen accumulation in atrial tissues, Masson trichrome staining was performed in this study. AAs sections were fixed in 4% paraformaldehyde and embedded in paraffin. The sections were subjected to Masson trichrome staining according to manufacturer's instruction and observed under microscopy (×400).

Atrial fibroblasts were fixed with 4% paraformaldehyde for 15 min, rinsed three times with PBS, and permeabilized with 0.1% Triton X‐100 for 15 min. Cells were blocked with 5% bovine serum album for 30 min at room temperature. Then, cells were incubated with the primary antibodies against either p53 or Smad3 at 4°C overnight. Subsequently, cells were incubated with the corresponding Alexa Fluor® conjugated secondary antibody for 2 h at room temperature in the dark. The secondary antibody solution was decanted, and cells were washed three times with PBS. Cells were incubated with DAPI for 10 min in the dark and images were captured under a laser confocal microscope.

### 2.13 Statistical analysis

Data are presented as mean ± SEM. Statistical analysis was performed with SPSS20.0. Differences between two groups were assessed by unpaired two‐tailed student's t test. Chi‐square test was used to compare AF inducibility. For multiple group comparisons, one‐way analysis of variance (ANOVA) was used followed by the Bonferroni correction. P value of <0.05 was considered statistically significant. All graphics were performed by GraphPad Prism 8.0. The number of samples, experiments, and animals per group are indicated in the figure legends.

## 3 RESULTS

### 3.1 Expression of p300, senescence, and fibrosis‐associated proteins were increased in left atrial appendages (LAAs) of AF patients and senescent human atrial fibroblasts

The expression values of all genes were derived from each atrium or atrial appendage samples in four independent affymetrix human gene expression microarrays (for accession number GSE1115574, GSE79768, GSE14975, and GSE41177). A detailed description of the GEO datasets was summarized in Table S1 in supplementary. We merged four datasets and extracted the expression values of corresponding genes for statistical analysis.

**FIGURE 1:**
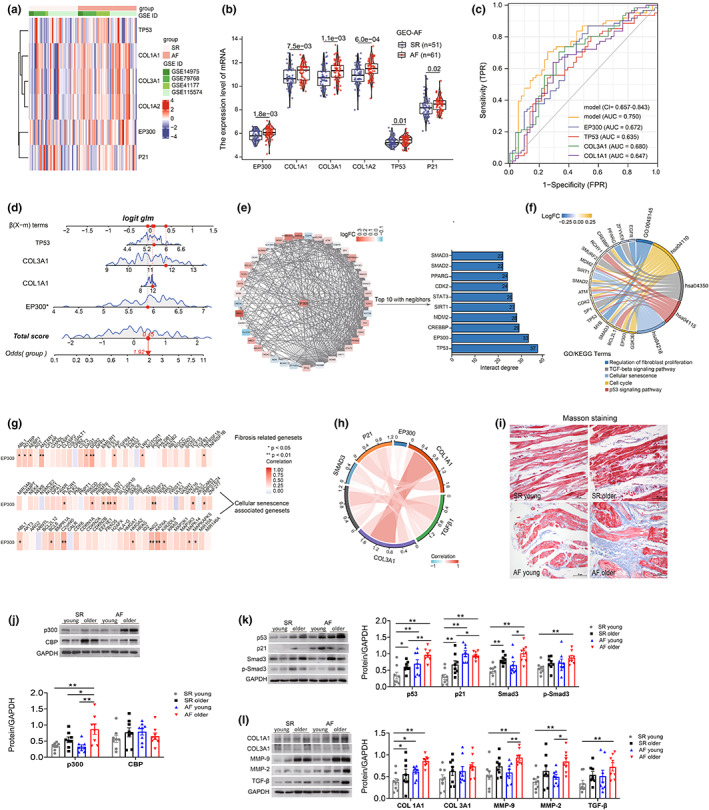
The expression differences of EP300/p300, aging, and fibrosis factors in atrial tissues of SR and AF patients were analyzed by bioinformatics analysis and were confirmed in LAAs of SR and AF patients with different ages. (a) Heatmap showed the expression profile of EP300 and its associated genes in AF and SR groups. (b) The scatterplot and violin‐boxplot illustrated the differences in gene expression between the two groups (AF vs. SR). (c) Receiver operating characteristic (ROC) analysis of individual EP300, COL1A1/3A1, and TP53 in merged datasets was presented. Clinical diagnostic power of four combined genes was also estimated (AUC = 0.750, 95% CI = 0.657–0.843) (logistic regression model = −16.440 + 1.188 * EP300 + 0.581 * TP53 + 0.514 * COL3A1 + 0.074 * COL1A1). The abscissa represented sensitivity and the ordinate represents 1‐specificity. Area under the ROC curve (AUC) represented the overall prediction performance in AF group. (d) Nomogram model for predicting the probability of AF based on four genes (EP300, TP53, COL1A1, and COL3A1). Red arrows represented example data, * indicated the value of EP300's clinical utility was statistically significant. (e) EP300 gene's interaction network diagram. Colors indicated the gene expression levels, the redder the color of the gene, the higher the gene expression level, and vice versa the bluer the color, the lower the expression level. A bar plot presented the top 10 high‐degree proteins and its interaction degree. (f) In the chord diagram, the biological processes and KEGG signaling pathways involved in EP300 and its related genes were visualized. (g) Correlation analysis between EP300, fibrosis gene sets, and cellular senescence gene sets. Red color represented positive correlation whereas blue represented negative correlation. *p < 0.05, **p < 0.01. (h) The correlation analysis of relevant genes involved in this study was performed in AF group (data from 61 AF samples). (i) Representative Masson staining of atrial specimens from patients with AF or SR controls of different ages. Scale bars, 50 μm. (j–l) Representative immunoblots and densitometric analysis of p300/CBP, senescence, and fibrosis‐associated proteins in LAAs from AF (*n* = 16) and SR (*n* = 16) patients with different ages, GAPDH was used as an internal control. *p < 0.05, **p < 0.01; data are mean ± SEM.

By analyzing the four different available microarray datasets of atrial tissues generated from AF patients and SR controls, we explored EP300(p300) senescent signal pathway TP53(p53)/p21 and fibrosis‐related gene expression between AF and SR patients. The results indicated that EP300, TP53, P21, and COL1A1/3A1 genes were highly expressed in the atrial of AF patients (Figure 1a,b). To further determine the diagnostic value of those genes, receiver operating characteristic (ROC) analysis of EP300, COL1A1/3A1, and TP53 was conducted. Results implied that all these genes had general ability to discriminate between AF and SR patients (EP300, AUC = 0.672; TP53, AUC = 0.635; COL1A1, AUC = 0.647; COL3A1, AUC = 0.680; Figure 1c). To improve the capacity in distinguishing AF patients from all samples, we constructed the combined diagnosis model of four crucial genes (EP300, COL1A1/3A1, and TP53), and the AUC value of the AF arrived at 0.750 (95% CI:0.657–0.843, Figure 1c). In order to improve the clinical applicability of the model, a nomogram was established. The results showed that EP300 was a risk factor for AF, the higher expression level of EP300, the higher incidence of AF (Figure 1d). EP300's co‐expression pattern was illustrated by Cytoscape. EP300 was located at the center of the network, co‐expression genes were arranged according to their interaction scores with EP300. Furthermore, bar plot presented the top 10 high‐degree proteins and its interaction degree (Figure 1e). Chord plot displayed the biological function and signaling pathway involved in EP300's co‐expression genes, the color depth represented the level of gene expression (Figure 1f). Regulation of fibroblast proliferation, TGF‐β signaling pathway, cellular senescence, cell cycle, and p53 signaling pathway were included in these results. We learned that cell senescence and fibroblast activation may be regulated by EP300 and its interacting genes. Thus, we explored an analysis of the pair‐wise correlation between three variables (EP300, fibrosis genesets, and cellular senescence genesets) in AF groups. The results showed EP300 was positively correlated with the above two gene sets in general (Figure 1g). Most of the genes in the above two gene sets were also positively correlated (Figure S1). Furthermore, the chord plot showed the level of association between EP300 and genes studied. (Figure 1h).

To prove the findings in the transcriptomic data analysis, we detected the atrial fibrosis and the protein expression levels of p300/CBP, p53/p21, Smad3/p‐Smad3, and fibrosis factor in atrium tissue of young and old SR/AF patients. As shown in Figure 1i, compared with SR patients, young and older AF patients showed increased atrial fibrosis detected by Masson staining. Meanwhile, the older AF patients exhibited higher p300 expression level than the young SR, old SR, and young AF groups, while there were no significant differences in CBP (CREB‐binding protein, the paralogue of p300, shares highly similar structural and functional features with p300) expression (Figure 1j). At the same time, higher protein levels of p53 and p21 were observed in young and older AF and older SR patients than in young SR patients (Figure 1k). As for Smad3 and p‐Smad3, the key proteins of fibrosis signal pathway were increased with age. Significantly higher levels of Smad3 and p‐Smad3 were observed in the young and older AF patients compared with their corresponding SR control patients. Protein levels of COL1A1, MMP‐2/9, and TGF‐β were also significantly elevated in the aged AF patients (Figure 1l). Altogether, these results indicated that p300 might be associated with aging and fibrosis, which may contribute to aging‐related atrial fibrosis and cause AF.

**FIGURE 2:**
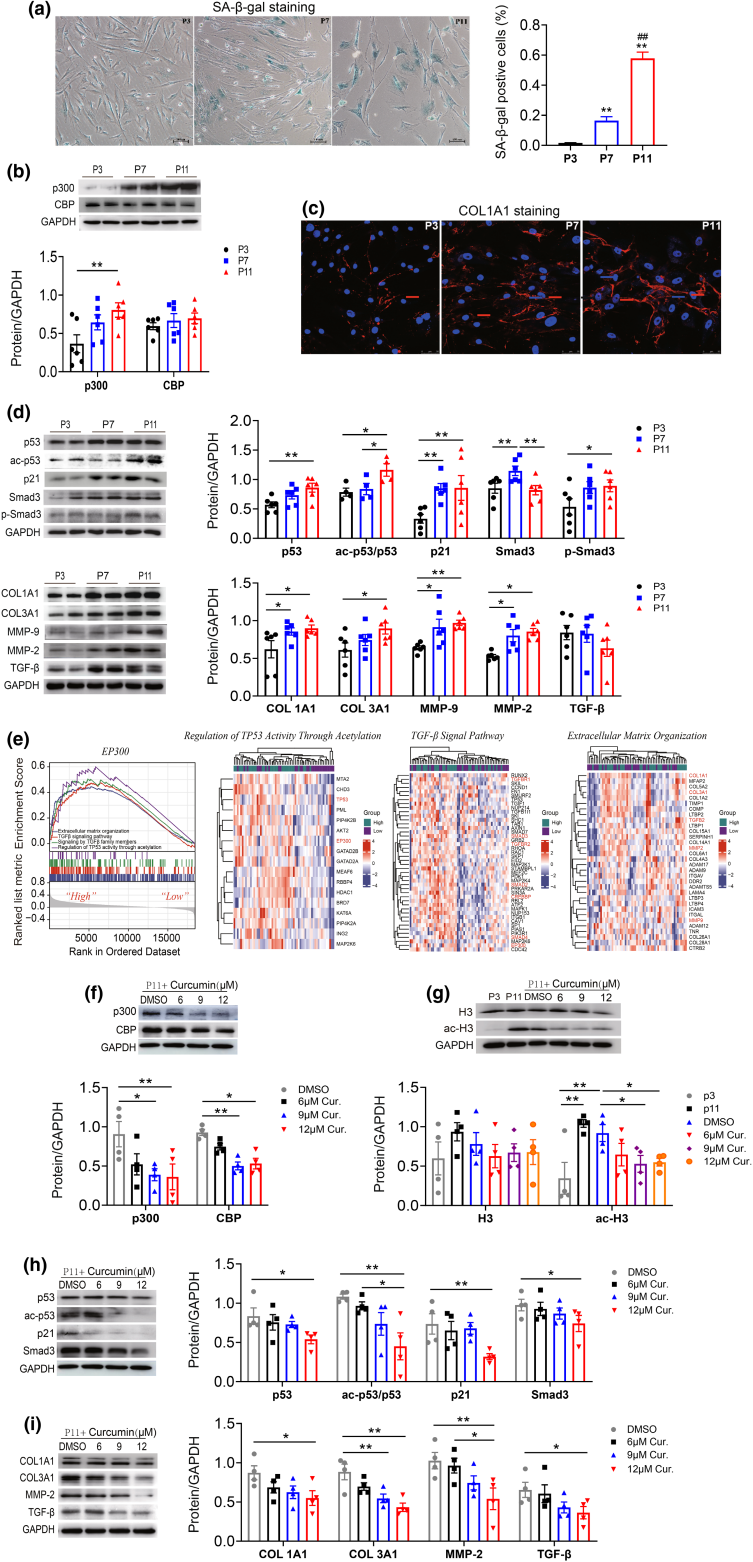
Expression of p300, senescence, and fibrosis‐associated proteins were increased in senescent human atrial fibroblasts and the effect of curcumin. (a). Representative SA‐β‐gal staining of human atrial fibroblasts of passage 3, 7, and 11. Scale bars, 100 μm. (b) Representative immunoblots and densitometric analysis of p300 and CBP in human atrial fibroblasts of different passage (P3, P7, and P11). *n* = 4–6. (c) Representative immunofluorescence staining of COL1A1 protein in human atrial fibroblasts of different passage (P3, P7, and P11). Scale bars, 100 μm. (d) Representative immunoblots and densitometric analysis of p53/ac‐p53, p21, and fibrosis‐associated proteins in human atrial fibroblasts of different passage (P3, P7, and P11). *n* = 4–6. (e) The potential biological function of EP300 determined by bioinformatics analysis. The results of Gene set enrichment analysis (GSEA) and the significantly enriched genes in the term of regulation of TP53 activity through acetylation, TGF‐β signal pathway, and extracellular matrix organization displayed by Heatmap. (f‐i) Representative immunoblots and densitometric analysis of p300/CBP, p53/ac‐p53/p21, H3/ac‐H3, and fibrosis‐associated proteins in senescent human atrial fibroblasts (P11) treated with 6, 9, or 12 μM curcumin. *n* = 4. *p < 0.05, **p < 0.01; Data are mean ± SEM.

Human atrial fibroblasts were isolated from left atrial tissue of SR patients and cultured to passage 11 (P11) to establish the senescent cells model. The SA‐β‐gal staining indicated that the senescence cells were markedly increased in P11 cells (Figure 2a). Compared to P3 cells, p300 protein levels were significantly increased in P11 human atrial fibroblasts, while CBP expression was not changed (Figure 2b). Senescence‐associated signal pathway p53, acetylation of p53 (ac‐p53/p53) and p21 expression levels were increased in P11 atrial fibroblasts compared to cells in P3 (Figure 2d). Moreover, the levels of profibrogenic proteins p‐Smad3, Smad3, and fibrosis‐associated proteins, such as TGF‐β, COL1A1/3A1, and MMP‐2/9, were also increased in P11 atrial fibroblasts (Figure 2d). Immunofluorescence staining indicated that the protein expression of COL1A1 in atrial fibroblasts was significantly increased with the increase of cellular passage (Figure 2c). These results indicated that p300 might be associated with atrial fibroblast senescence and fibrosis‐associated protein expression.

### 3.2 p300‐mediated human atrial fibroblast senescence and fibrosis through p53/Smad3 pathway

To systematically assess the potential biological functions of EP300 in AF, GSEA was conducted to identify the differentially activated signaling pathways in the high EP300 expression group. Results showed that the term of extracellular matrix organization, TGF‐β signaling pathway, signaling by TGF‐β family members, and regulation of TP53 activity through acetylation were significantly enriched in high expression group of EP300. Heatmap showed that the significantly enriched genes in EP300 high group are mainly involved in the regulation of TP53 activity through acetylation, TGF‐β signal pathway, and extracellular matrix organization (Figure 2e).

### 3.3 Intervention of p300 affected the senescence and fibrosis factor secretion of atrial fibroblasts through regulating p53/Smad3

Subsequently, we explored the underlying mechanism mediating the promoting effect of p300 on atrial fibroblast senescence and fibrosis‐associated protein expression. Senescent human atrial fibroblasts (P11) were treated with different concentrations of curcumin (6, 9, or 12 μM), a natural polyphenol derived from Asian spice turmeric which inhibit p300 protein expression and its acetyltransferase activity. We found that both p300 and CBP protein expression were decreased, and the senescence‐associated proteins p53, ac‐p53/p53, p21, Smad3, p‐Smad3, and fibrosis proteins COL1A1, MMP‐2/9, TGF‐β expression levels were reduced in higher concentration of curcumin treatment groups (9 or 12 μM) (Figure 2f,h‐i). In addition, compared with young cells (p3), the protein expression of ac‐H3 was also increased in senescent human atrial fibroblasts (p11), which could be improved by curcumin (Figure 2g), without the change of H3 protein expression. Similar results were found in senescent cells treated with selective histone acetyltransferase p300 inhibitor, C646 (Figure S3). Previous study has revealed that p300 promotes myocardial fibrosis by mediating acetylation of Smad in diabetic rats or hypertensive mice (Bugyei‐Twum et al., 2014; Rai et al., 2019). Therefore, we draw a conclusion that p300 may up‐regulate p53 protein and acetylation levels, then participate in the modulation of senescence and fibrosis‐relative factors.

**FIGURE 3:**
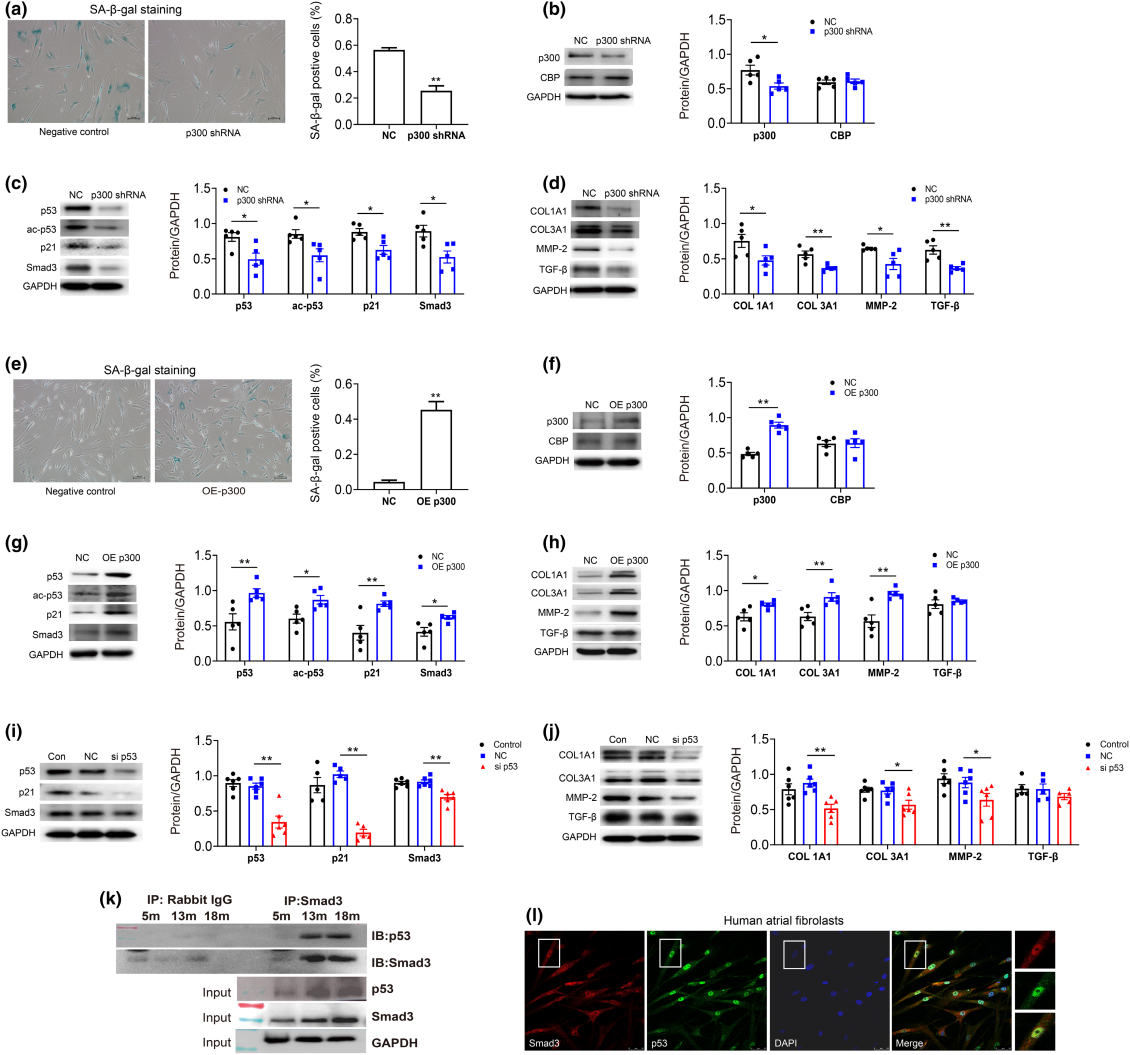
p300‐mediated atrial fibroblast senescence and fibrosis signals through p53/Smad3 pathway in human atrial fibroblasts. (a‐d) Representative SA‐β‐gal staining of senescent human atrial fibroblasts (p11) treated with or without p300shRNA. Scale bars, 100 μm. Representative immunoblots and densitometric analysis of p300/CBP, senescence, and fibrosis‐associated proteins in aged human atrial fibroblasts (P11) treated with p300 shRNA. (e‐h) Representative SA‐β‐gal staining of young cells (p3) treated with or without p300 overexpression. Scale bars, 100 μm. Representative immunoblots and densitometric analysis of p300/CBP, senescence, and fibrosis‐associated proteins in young human atrial fibroblasts (P3) with p300 overexpression. (i and j) Representative immunoblots and densitometric analysis of p53/p21, Smad3, and fibrosis‐associated proteins in aged HAFs (P11) treated with p53 siRNA. *p < 0.05, **p < 0.01; data are mean ± SEM. (k) Co‐immunoprecipitation of p53 with Smad3 in atrial tissue from 5 m, 13 m, and 18 m mice. Immunoblots for p53 (top) and Smad3 (bottom) from samples exposed to protein a/G beads coated with rabbit IgG (negative control) or p53 antibody. (l) Co‐localization of p53 with Smad3 confirmed by confocal microscopy. Human atrial fibroblasts were co‐stained with anti‐p53 antibody, anti‐Smad3, and DAPI. Merged images of p53 (green), Smad3 (red), and DPAI (blue) are shown in the insert. Scale bar represents 100 μm.

CBP shares structural and functional features with p300, the decreased expression of CBP was also detected in atrial fibroblasts treated with curcumin. To eliminate the role of CBP, p300 shRNA was used to inhibit p300 gene expression in passage 11 human atrial fibroblasts. As shown in Figure 3a–d p300 knockdown significantly decreased ac‐p53, p53, and p21 protein expression and the ratio of SA‐β‐gal‐positive senescent cells. Moreover, the expression levels of profibrotic protein Smad3 and p‐Smad3, as well as fibrosis‐related proteins Col1A1, MMP‐2/9, TGF‐β, were also reduced. Conversely, overexpression of p300 using p300 plasmid increased the ratio of SA‐β‐gal‐positive cells and upregulated the protein expression of ac‐p53, p53, Smad3, and fibrosis factors in young human atrial fibroblasts (P3) (Figure 3e–h). In summary, these results suggest that p300 activates senescence‐related fibrosis through p53/Smad3 pathway.

### 3.4 p53 regulated senescent atrial fibroblasts fibrosis signals expression through p53/Smad3 complex formation

As we all known, p53/p21 is one of the classic senescence signaling pathways, it also has been reported that p53 is associated with tissue fibrosis and extracellular matrix production. As shown in Figure 3i,j, p53 knockdown by siRNA significantly inhibited p21, Smad3, p‐Smad3, COL1A1/3A1, MMP‐2/9, and TGF‐β expression in human atrial fibroblasts.

It has been reported that p53 interacts with Smad2/3 to affect fibrosis‐related protein expression, we thus examined whether p53 interacted with Smad3 to form protein complex in regulating the expression of fibrosis factors in human atrial fibroblasts. Co‐immunoprecipitation technique was used to assess the potential interaction of p53 with Smad3 in mouse atrial tissues (Figure 3k). The Smad3 was precipitated using antibody‐coated beads, Western blots demonstrated the presence of p53 in the precipitated (Figure 3k). Furthermore, Smad3 and p53 were found mainly in nucleus of human atrial fibroblasts and were also observed in cytoplasm (Figure 3l). Their expressions overlap to some degree (insert of Figure 3l). The results suggest that p53 interacts with Smad3, and the p53/Smad3 protein complexes increase with aging.

### 3.5 Curcumin treatment decreased the senescence and fibrosis of atrial tissue and AF inducibility in aged mice

**FIGURE 4:**
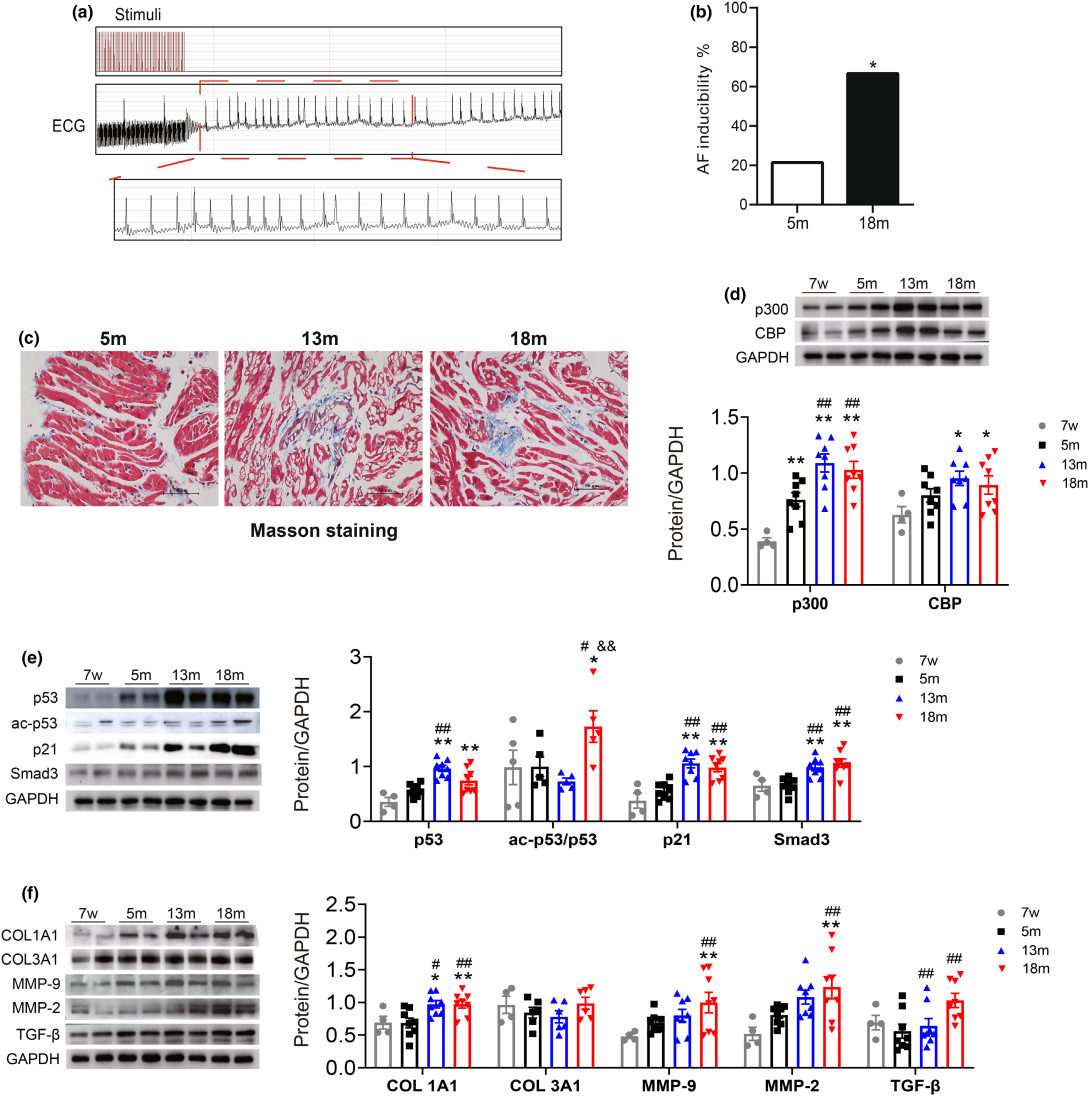
AF inducibility and the senescence and fibrosis‐associated proteins in atrial tissue of C57BL/6 mice with different ages. (a). Surface electrocardiogram (ECG) showing that AF was successfully induced by atrial burst stimulation in mice. (b) The inducible rate of AF in mice with different ages. *n* = 12. *p < 0.05 vs. 5 m mice. (c) Representative Masson staining of atrial specimens from mice of different ages. Scale bars, 50 μm. (d–f). Representative immunoblots and densitometric analysis of p300/CBP, senescence, and fibrosis‐associated proteins in atrial tissues from mice with different ages. *n* = 6–8. *p < 0.05, **p < 0.01 vs. 7‐week mice; ^#^ *p* < 0.05, ^##^ *p* < 0.01 vs. 5 m mice. Data are mean ± SEM.

To further confirm the role of p300 in senescent‐related atrial fibrosis, studies were performed in aged mouse models. Baseline information of young and aged mice is shown in Table 2. Hair loss and myocardial hypertrophy occurred in elderly mice, which were characterized by increased heart weight‐to tibial length ratio and ventricular wall thickness (Figure S4). Electrophysiological parameters are displayed in Table 2. P‐wave duration (PWD) and PR interval were significantly prolonged in the aged group (18‐month mice) compared to the young group (5‐month mice), suggesting the conduction in intra‐atrial and atrioventricular was delayed. SNRT and CSRNT at a 100 ms S1S1 stimulation cycle length in 18‐month mice were also increased significantly compared to the young group. All these electrophysiological changes contributed to the increased vulnerability of AF in aged mice. The induction rate of AF in the elderly group was significantly increased compared to that in the young group (Figure 4a,b).

**TABLE 2:** Anatomic, electrophysiology, and functional data (5 and 18 m)

	5 m	18 m
*n*	12	12
Body weight (g)	25.97 ± 0.68	32.18 ± 0.50**
Heart wt/TL (mg/mm)	7.75 ± 0.37	8.70 ± 0.33
HR (bpm)	529.33 ± 15.85	595.04 ± 8.44**
PWD (ms)	18.92 ± 0.66	20.50 ± 0.89
PR interval (ms)	44.42 ± 2.44	52.25 ± 2.40*
SNRT (ms)	132.42 ± 3.18	166.42 ± 8.33**
CSNRT (ms)	23.68 ± 1.58	52.18 ± 5.32**
Total AF duration (s)	6.72	113.80
LVEDD (mm)	3.30 ± 0.11	3.64 ± 0.11*
LVESD (mm)	2.35 ± 0.13	2.65 ± 0.15
LVd (μl)	44.89 ± 3.51	56.90 ± 4.04*
LVs (μl)	20.17 ± 2.61	27.40 ± 4.33
LVEF (%)	56.95 ± 2.89	54.13 ± 3.64
LVFS (%)	29.33 ± 1.82	27.83 ± 2.12

*Note*: Data are mean ± SEM.

Abbreviations: AERP, atrial effective refractive period; CSNRT, corrected SNRT; HR, heart rate; LVd, left ventricular diastolic volume; LVEDD, left ventricular end‐systolic dimension; LVEF, left ventricular ejection fraction; LVESD, left ventricular end‐diastolic dimension; LVFS, left ventricular fractional shortening; LVs, left ventricular systolic volume; PWD, p wave duration; SNRT, sinus node recovery time.

* *p* < 0.05 vs. 5 m

** *p* < 0.01 vs. 5 m.

Masson staining showed that atrial fibrosis increased in aged mice (Figure 4c). The expression levels of p300, senescence, and fibrosis‐related protein in mouse atrial tissues were also detected. As shown in Figure 4d–f, 13‐ and 18‐month‐old mice showed increased protein level of p300, senescence‐associated proteins p53, ac‐p53, p21, and fibrosis‐related proteins COL1A1, MMP‐2/9, TGF‐β, Smad3, and p‐Smad3 compared to 7‐week or 5‐month‐old mice. These results were consistent with those obtained from human LAAs, indicating that elevated p300 might be involved in aging and aging‐related fibrosis.

**FIGURE 5:**
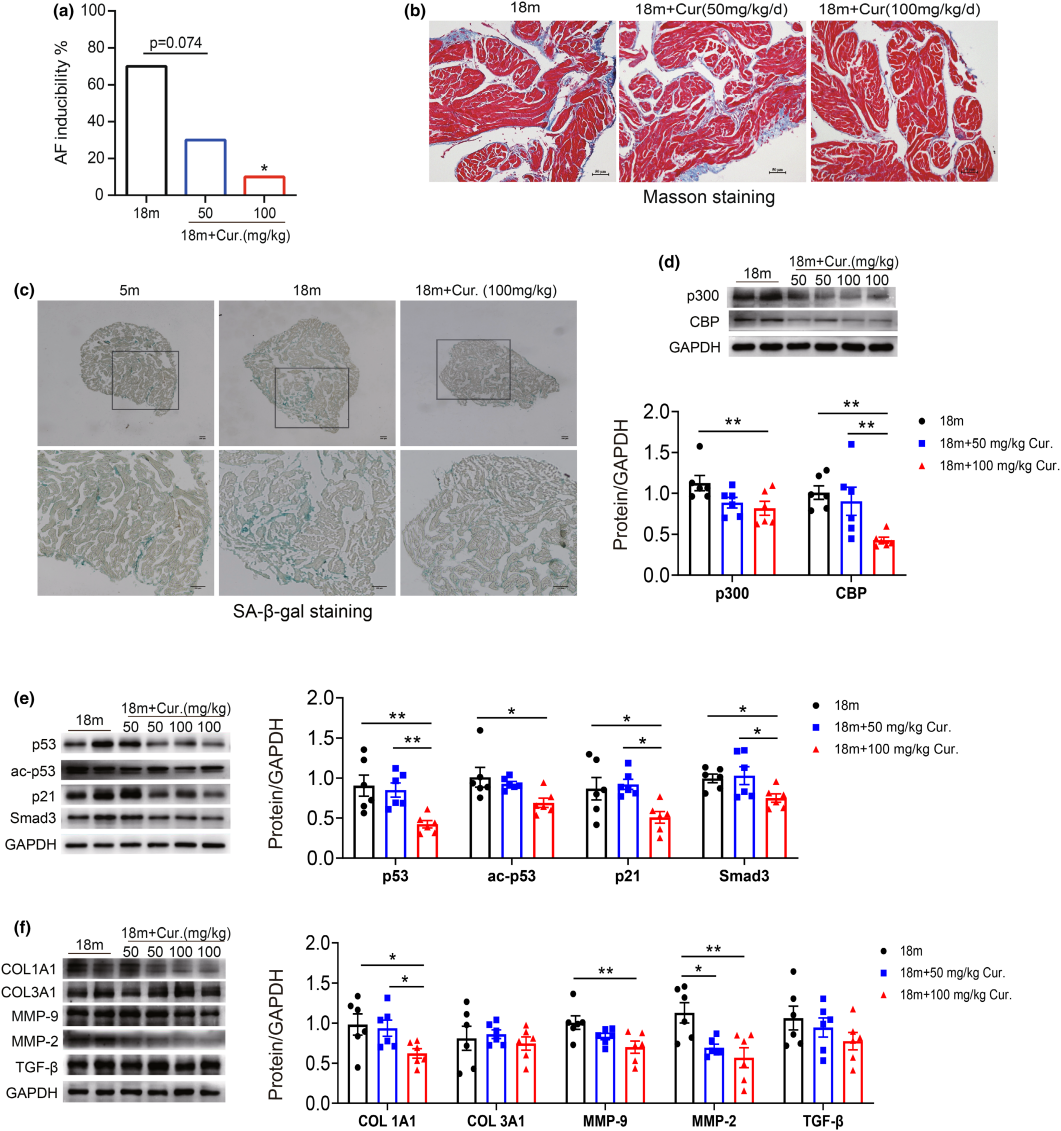
The role of curcumin in regulation of senescence and fibrosis‐associated proteins and AF inducibility in atrial tissue of aging mice. (a) The inducible rate of AF in mice treated with different dosage of curcumin. *n* = 10, *p < 0.05, vs. 18 m mice; (b) Representative Masson staining of atrial specimens obtained from aged (18 m) mice treated with different doses of curcumin (50 or 100 mg/kg/d). Scale bars, 50 μm. (c) Representative SA‐β‐gal staining of atrial tissue obtained from young (5 m) and aged (18 m) mice with or without curcumin treatment (100 mg/kg/d). Scale bars, 100 μm. (d–f) Representative immunoblots and densitometric analysis of the expression of p300/CBP, senescence, and fibrosis‐associated proteins in atrial tissues obtained from curcumin‐treated mice. *n* = 6–8. *p < 0.05, **p < 0.01; data are mean ± SEM.

Then, we treated aged mice (18 m) with curcumin (50 or 100 mg/kg/day) for 6 months starting at 12 months of age. Anatomic, electrophysiology, and functional data of the mice were shown in Table S2. As shown in Figure 5a, high doses of curcumin (100 mg/kg/day) could improve electrophysiologic characteristics and reduce AF susceptibility of aged mice. The SA‐β‐gal staining indicated that 100 mg/kg curcumin could ameliorate the senescence of atrial tissue (Figure 5b). The fibrosis degree of mice atrium was also reduced in curcumin (100 mg/kg/day) treated group (Figure 5c). Quantification of immunoblot signals revealed that treatment with 100 mg/kg curcumin significantly reduced the protein expression level of p300 and CBP in the atrial tissue of 18‐month mice (Figure 5d). Moreover, the protein expression of p53, ac‐p53 and p21 were also significantly decreased as well as the p‐Smad3, Smad3, MMP‐2 and COL1A1 in mice treated with curcumin (100 mg/kg/day) (Figure 5e,f). These results demonstrated that inhibition of p300 rescued senescence and fibrosis in aged mice, as well as AF vulnerability.

### 3.6 AF induction and aging‐related atrial fibrosis were decreased in aged Cre + p300 (+/−) mice

**FIGURE 6:**
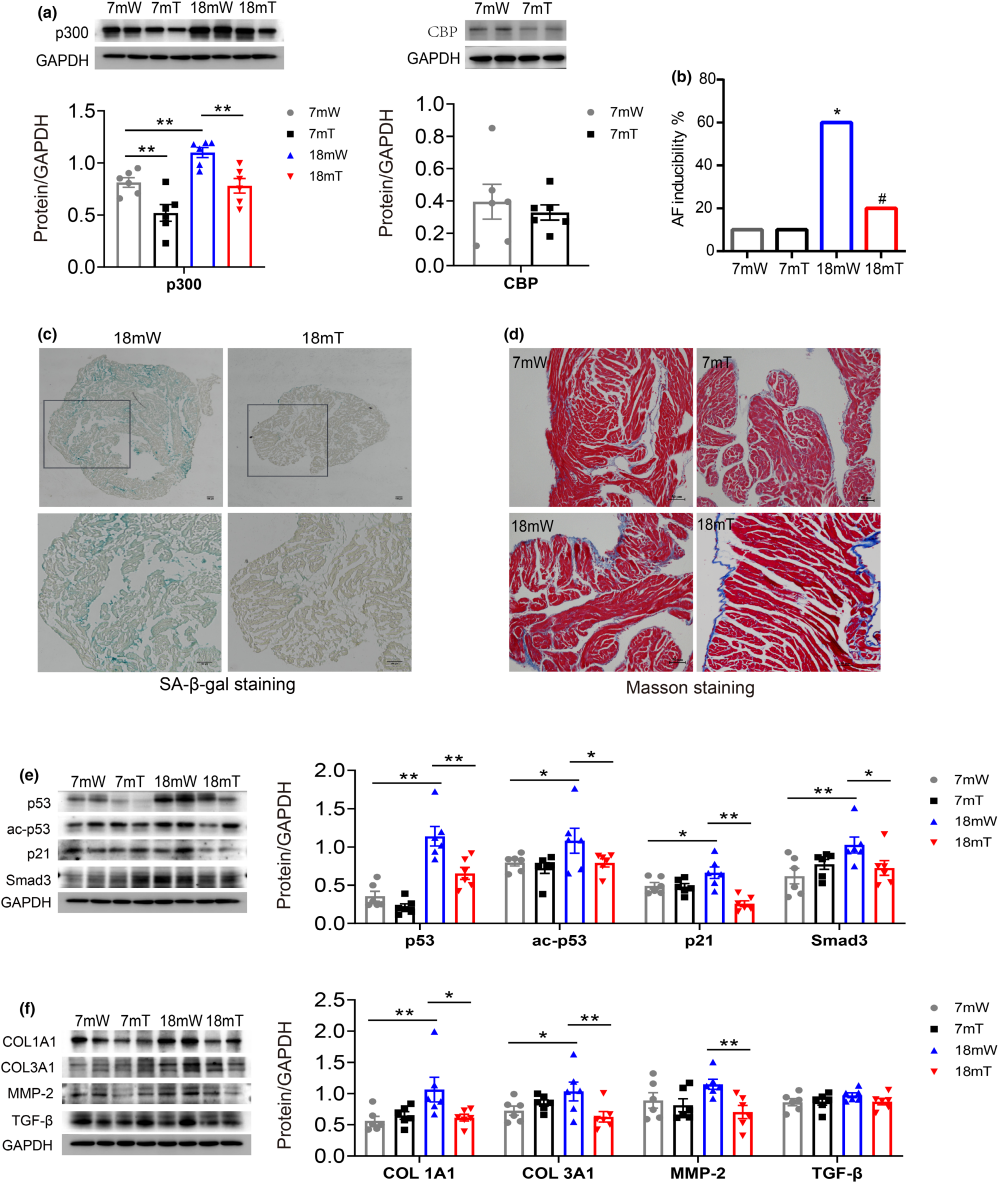
The inducible rate of AF and the age‐related atrial fibrosis were decreased in Cre + p300 (+/−) (het) mice. (a) Representative immunoblots and densitometric analysis of p300 and CBP in atrial tissues obtained from young (7 m) and aged (18 m) p300 (+/+) (wt) and Cre + p300 (+/−) (het) mice. *n* = 6. *p < 0.05, **p < 0.01; data are mean ± SEM. (b) The inducible rate of AF in 18 m p300 (+/+) (wt) and Cre + p300 (+/−) (het) mice. *n* = 10, *p < 0.05, vs. 7 mW, ^#^ *p* < 0.05, vs. 18 mW; (c) Representative SA‐β‐gal staining of atrial tissue from aged (18 m) p300 (+/+) (wt) and Cre + p300 (+/−) (het) mice. Scale bars, 100 μm. (d) Representative Masson staining of atrial specimens from 18 m p300 (+/+) (wt) and Cre + p300 (+/−) (het) mice. Scale bars, 50 μm. (e and f) Representative immunoblots and densitometric analysis of senescence and fibrosis‐associated protein expression in atrial tissues obtained from young (7 m) and aged (18 m) p300 (+/+) (wt) and Cre + p300 (+/−) (het) mice. *n* = 6. *p < 0.05, **p < 0.01; data are mean ± SEM.

To confirm p300, but not CBP, is essential for aging‐related fibrosis and AF induction, Cre + p300 (+/−) (Het) mice [Systemic p300 (−/−) homozygotes are prone to embryonic death] was employed (The genotype identification was shown in Supplemental Methods and Figure S5) (Figure 6a). We found that compared to 18‐month p300 (+/+) (wt) mice, electrophysiologic characteristics were improved and AF susceptibility was reduced in Cre + p300 (+/−) (Het) mice at the same age (Table S3 and Figure 6b). The ratio of senescence (Figure 6c) and atrial fibrosis (Figure 6d) was also improved in aged Cre + p300 (+/−) (Het) mice. Moreover, the protein expression of p53, ac‐p53, p21, and Smad3 were remarkedly reduced as well as the fibrosis‐related protein COL1A1/3A1 and MMP2 in atrium tissue of 18‐month Cre + p300 (+/−) (Het) mice (Figure 6e,f). These indicated that p300, but not CBP, is involved in the age‐related atrial fibrosis which contributes to the AF occurrence.

## 4 DISCUSSION

The prevalence of AF increases with age. Numerous studies have reported that atrial fibrosis increases with age, which is considered as the possible pathological mechanism of AF in the elderly. However, the precise mechanism of aging‐related atrial fibrosis remains unclear. In this study, by using bioinformatics analysis, we found that p300, senescence, and fibrosis markers were highly expressed in AF group, and more importantly, the co‐expression and co‐localization between p300 and senescence markers were observed through PPI network. The correlation analysis also showed a general correlation between them. These results indicated that p300 may involve in the biological process of cellular senescence and fibrosis. To verify the hypothesis, a series of experiments were carried out. In this study, we present evidence that p300 acts as a profibrotic factor in aging mice by promoting atrial fibroblast senescence and fibrosis‐related protein expression. The protein expression of p300 was increased in senescent atrial fibroblasts and aged atrial tissues in human and mice. Importantly, fibrosis factors, such as Collagen, MMP‐2/9, TGF‐β, were also upregulated in senescent atrial fibroblasts. Mechanistically, we assessed that p53/Smad3 was the potential signaling pathway of the p300's profibrotic and pro‐senescence effect in atrial fibroblasts. These effects mainly due to the acetylation of p300 because ac‐p53 and ac‐H3 were significantly increased in senescent atrial fibroblasts, while inhibition of p300 can significantly reduce the acetylation activity in senescent cells. Further, inhibiting or knockdown of p300 or p53 rescued the atrial senescence and fibrosis and reduced AF susceptibility. Thus, we finally revealed that p300 might contribute to the aging‐related atrial fibrosis through p53/Smad3 axis and p300 can be a potential therapeutic target in delaying senescence and AF therapy.

Many animal studies have shown higher AF inducibility in aged group (Hayashi et al., 2002; Luo et al., 2013). Recently, a study showed that atrial appendage from patients with permanent AF displayed higher expression of p53 and p21 than that from SR patients, indicating that atrial senescence was closely related to AF development (Jesel et al., 2019). In line with the report, we found elevated senescence‐related proteins p53, p21 in elder AF patients and higher AF inducibility in aged mice. In addition, atrial fibrosis is critically involved in AF initiation, progression, and maintenance. Many factors influence atrial fibrogenesis during the development of AF, including aging. Increased atrial fibrosis was observed either in elder patient or aged animal group in this study. Meanwhile, PDW and PR interval were prolonged in aged mice, which to some extent reflected atrial structural remodeling (Huo et al., 2014; Josephson et al., 1977). Prolonged SNRT/CSNRT in aged mice was consistent with previous studies (Dun & Boyden, 2009; Jansen et al., 2017). We assessed the phenomenon that atrial fibrosis increased with age, and aging‐related atrial fibrosis is one of the possible pathogeneses of AF in the elderly. However, the potential mechanism of aging‐related atrial fibrosis remains unclear.

Some studies showed co‐transcriptional activator, and acetyltransferase p300, as the opposite regulator of histone deacetylase, is involved in aging or senescence (Ghosh, 2020). A recent study confirmed p300 as a driver of senescence via inducing super‐enhancers in senescent cells, and p300 depletion delayed cell senescence, but not the paralogous CBP (Sen et al., 2019). Studies from cellular and animal models have indicated the pivotal role of p300 in accelerated cardiac aging pathologies including cardiac hypertrophy and matrix remodeling. Suppression of p300 acetyltransferase activity by natural molecules (curcumin) or synthetic small molecule inhibitors of p300 (L002 or C646) leads to slowdown or reversal of cardiac aging pathologies induced by hypertension, diabetes, and other stress (Rai et al., 2017, 2019). Conversely, some studies found that C646 inhibited human melanoma cell growth, and accelerated cell senescence (Yan et al., 2013). p300 robustly increased lifespan in flies and mice acting as an inhibitory target of nordihydroguaiaretic acid (NDGA) which is a life span increasing drug (Tezil et al., 2019). Thus, the role of p300 in aging is controversial. In addition, elevated levels of p300 are associated with cardiac fibrosis. For example, p300 might promote myocardial fibrosis, by mediating acetylation of Smad, during the pathological hypertrophy in diabetic rats or hypertensive mice (Bugyei‐Twum et al., 2014; Rai et al., 2019). But few studies have focused on the role of p300 in the atrial fibrosis induced by aging. In this study, we observed that p300 was upregulated and accompanied by senescence and fibrosis in elder AF patients as well as aged mice. Curcumin treatment and half p300 knockdown reduced atrial senescence, fibrosis, even AF inducibility in aged mice. It provides evidence that p300 has promoting effect on aging process and aging‐related atrial fibrosis, further affecting AF development.

Cell senescence is a hallmark of organismal aging, and senescent cell accumulation is usually considered as a driver of aging (He & Sharpless, 2017). Fibroblasts, considered as the main senescent cell type in aging heart, are the central effector in fibrosis. In the present study, we established a cell aging model through cell passage culture and found that human atrial fibroblasts at higher passage (p11) showed increased SA‐β‐gal activity, elevated p300, senescence, and fibrosis‐related proteins and p300 inhibition in which reversed cell senescence and reduced fibrosis‐related protein expression. It is likely that p300 regulates atrial fibroblast senescence to participate in aging‐related fibrosis and finally promotes AF development and progression.

Besides its direct role in chromatin remodeling and transcriptional activation of targeted gene regulatory domains, acetyltransferase p300 is also involved in crosstalk of cellular signaling pathways to control several cellular functions. Smads are a family of proteins that play important roles in the context of fibrosis, in which Smad3 is one of the profibrotic molecules (Zhang, 2018). Interestingly, p53 and Smad3, which are the key components of cell senescence and fibrosis signaling pathway, respectively, have been reported as the downstream targets of p300 (Vaziri & Benchimol, 1999). In current study, we observed the link between increased p53, ac‐p53, Smad3, pSmad3 protein expression and the upregulation of p300 in atrial fibroblast senescence and fibrosis‐related protein expressions. Furthermore, inhibition of p300 protein significantly suppressed p53, ac‐p53, Smad3, pSmad3 protein expression and reduced senescence and fibrosis in fibroblasts at higher passage and aged mice atrial tissues. Conversely, overexpression of p300 in low passage (P3) increased the ratio of senescent cells and the expression of fibrotic cytokines. Given that p53 can regulate collagen expression by influencing cardiac fibroblast senescence (Zhu et al., 2013), and p53/Smad3 axis is the new target for renal fibrosis therapy (Overstreet et al., 2014), we further explored the potential role of p53/Smad3 in atrial fibrosis senescence and fibrosis‐related protein expression. Knockdown of p53 reduced fibrosis‐related protein expression in senescent human atrial fibroblasts. Together with previous studies, p53 and Smad3 might synergistically regulate fibrosis‐related protein expression in senescent atrial fibroblasts. As expected, p53/Smad3 complex was detected, indicating that p53/Smad3 complex formation played a role in promoting aging‐related atrial fibrosis.

In conclusion, although much attention has been paid to the association between aging and cardiac fibrosis, the mechanism underlying aging‐related atrial fibrosis has not yet been well elucidated. In this study, we verified that p300 is a novel target for AF treatment. Mechanistically, p300, through p53/Smads signaling pathway, promotes atrial fibroblast senescence and fibrosis‐related protein expression and leads to AF development. Inhibition of p300 significantly rescued atrial fibroblast senescence and aging‐related atrial fibrosis.

## AUTHOR CONTRIBUTIONS

FR, S‐L W, and C‐Y D designed the study. X‐Y G, Y‐Y L, X‐S L, D‐W P, Q‐Q L, H‐S Z, H‐M G, J‐F Z, H Y, S‐J K, Z‐Y W, M‐Z Z conducted the experiments and acquired the data. FR, X‐Y G, Y‐Y L, and Q‐Q L performed data analysis. FR, S‐L W, C‐Y D, X‐Y G, Y‐Y L, Q‐Q L, and Y‐M X wrote and revised the manuscript. All authors contributed to the article and approved the submitted version.

## FUNDING INFORMATION

This work was supported by the National Natural Science Foundation of China (No. 81670314 to F.R., and 81870254 to Y.M.X.), High‐level Hospital Construction Plan (No. DFJH201808 to S.L.W. and DFJH201925 to H.Y.) and the Guangzhou Municipal Science and Technology Project (201804010059 to F.R. and 202102080385 to C.Y. D.).

## CONFLICT OF INTEREST

The authors declare that they have no competing interests.

## Supporting information


Appendix S1

## Data Availability

The data that support the findings of this study are available from the corresponding author upon reasonable request. Expended methods can be found in the Online Supplemental Methods and Data.
