# Supplementary material for: Acetyltransferase p300 regulates atrial fibroblast senescence and age‐related atrial fibrosis through p53/Smad3 axis
Source: Aging Cell. 2022 Dec 5;22(1):e13743. doi: 10.1111/acel.13743 (PMC9835568; doi:10.1111/acel.13743)
Supplement: Supplementary file 1 — Appendix S1 [file ACEL-22-e13743-s001.docx]

**Supplement to Methods section**

**Generation and determination of p300 -/+ mice:** Studies were conducted in mice on a C57BL/6J background. Germline, mice heterozygous for a targeted p300 allele (p300-/+) were generated by crossing p300 floxed mice (the kind gift of Prof. Nanette H. Bishopric and Dr. Jianqin Wei) with CAG-cre mice (B6-CAG-Cre, a mouse model of systemic expression of CRE enzyme driven by CAG promoter; Jiangsu GemPharmatech Co., Ltd, China). Floxed, but Cre negative, littermates were used as experimental controls; these mice are referred to as wild type (WT). Animals were genotyped at one week old using the polymerase chain reaction. The following primers were used to detect p300, null phenotype, and CAG-cre phenotype respectively: 1) p300: p300-F: TGG ACT GGT TAT CGG TTC ACC, p300-R:CAG TTA CAT ACA GCT GTG ATG, the product: WT:800bp, mut:1000bp; 2) null phenotype: p300-F: TGG ACT GGT TAT CGG TTC ACC, p300-R1: GCA ACT GTT CAA GGT AAG TCA CAC, the product, Flox：2181 bp, WT：1981 bp, Null：~500 bp; 3) CAG-cre phenotype: Rabbit-pA-tF1: CCT GCT GTC CAT TCC TTA TTC CAT A, H11-tR3: ATA TCC CCT TGT TCC CTT TCT GC, the product, T：337bp.

**Electrophysiological parameters:** Sinus node recovery time (SNRT) was measured after a 30s pacing train on the right atrium with 100ms basic cycle length (BCL), 1ms pulse width and a stimulus amplitude of 2-fold diastolic capture threshold. The atrial effective refractory period (AERP) was determined by a series of regular pacing trains at BCL of 100ms, 80ms, 60ms, with a coupled shorter S2 premature stimulus. AERP was defined as the longest S1-S2 interval that failed to generate a propagated beat with S2.

**Antibodies used in this study.**

| Antibodies | Source |  |
| --- | --- | --- |
| Mouse anti-p300 CT (1:1000) | EMD Millipore | Cat#05-257 |
| Mouse anti-CBP (1:1000) | Santa cruz | Cat#sc-7300 |
| Rabbit anti-COL1A1 (1:2000) | Abcam | Cat#ab34710 |
| Rabbit anti-COL3A1 (1:2000) | Abcam | Cat#ab7778 |
| Rabbit anti-MMP-9 (1:1000) | EMD Millipore | Cat#AB19016 |
| Rabbit anti-MMP-2 (1:1000) | EMD Millipore | Cat#AB19015 |
| Mouse anti-p53 (1:1000) | Cell signaling | Cat#2524 |
| Rabbit anti-acp53 (1:1000) | Cell signaling | Cat#2570 |
| Mouse anti-Smad3 (1:1000) | Cell signaling | Cat#9523 |
| Rabbit anti-pSmad3 (1:1000) | Cell signaling | Cat#9520 |
| Rabbit anti-TGF-β (1:1000) | Cell signaling | Cat#3711 |
| Mouse anti-p21 (1:500) | Santa cruz | Cat#sc-817 |
| Rabbit anti-H3 (1:1000) | Cell signaling | Cat#9715 |
| Rabbit anti-acH3 (1:1000) | Cell signaling | Cat#9649 |
| AffiniPure Goat Anti-Mouse IgG (1:5000) | Jackson | Cat#115-035-003 |
| AffiniPure Goat Anti-Rabbit IgG (1:5000) | Jackson | Cat#111-035-003 |

**Drug:** Curcumin ( Cayman Chemical, Ann Arbor, Michigan, USA)

C646 (Merck, Darmstadt, Germany)

**For p300 knockdown of human atrial fibroblasts, the sequence are shown below:**

**Target sequences：**

1) EP300-RNAi-1: ccCGGTGAACTCTCCTATAAT

2) EP300-RNAi-2: ccAGCCTCAAACTACAATAAA

3) EP300-RNAi-3: cgAGTCTTCTTTCTGACTCAA

**shRNAs sequences:**

1）Ep300-RNAi-1-5’：GATCCCGCGGAATACTATCACCTCCTACTCGAGTAGGAGGTGATAGTATTCCGCTTTTTGGAT

2）Ep300-RNAi-1-3’：

GGCGCCTTATGATAGTGGAGGATGAGCTCATCCTCCACTATCATAAGGCGAAAAACCTATCGA

3）Ep300-RNAi-2-5’：

GATCCCGCTAGTCCTATGGGTGTAAATCTCGAGATTTACACCCATAGGACTAGCTTTTTGGAT

4）Ep300-RNAi-2-3’：

GGCGATCAGGATACCCACATTTAGAGCTCTAAATGTGGGTATCCTGATCGAAAAACCTATCGA

5）Ep300-RNAi-3-5’：GATCCCccAGTCCTTATGGTTCACCATCTCGAGATGGTGAACCATAAGGACTGGTTTTTGGAT

6）Ep300-RNAi-3-3’：

GGGGTCAGGAATACCAAGTGGTAGAGCTCTACCACTTGGTATTCCTGACCAAAAACCTATCGA

All the shRNAs were purchased from Shanghai Genechem Co.,Ltd, China.

**Human EP300 gene was overexpressed in human atrial fibroblasts using piggyBac technique, the CMV promoter is shown below:**

p300 CMV promoter: PB-CMV>hEP300-PGK>eGFP/T2A/Puro,

Control: PB-PGK>eGFP/T2A/Puro;

Template Needed, Human EP300 CDS: NM_001429.4 (7245 bp) (Cyagen Biosciences Inc , China)

**Three different siRNAs were used to knockdown p53 in human atrial fibroblasts and the sequence are shown below:**

1. hTP53 si-1 sense:GCGCACAGAGGAAGAGAAUTT
2. hTP53 si-2 sense:CCACUGGAUGGAGAAUAUUTT
3. hTP53 si-3 sense:CCAUCCACUACAACUACAUTT

Scrambled：TTCTCCGAACGTGTCACGT

All the siRNAs were purchased from Shanghai Genechem Co.,Ltd, China

**Supplemental Figures**

**
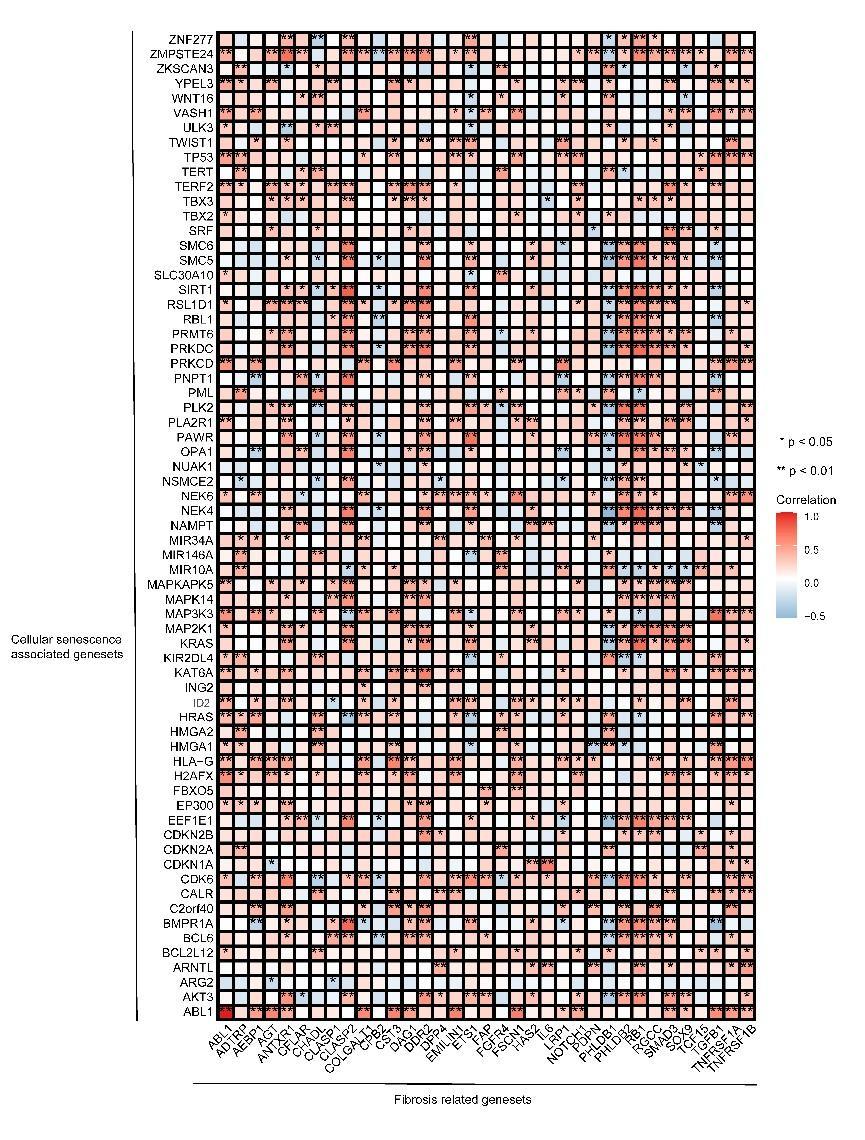
**

**Figure S1. Correlation Analysis of two phenotype-related genes in the AF group. The color red indicates a positive correlation, while the color blue indicates a negative correlation.**

**
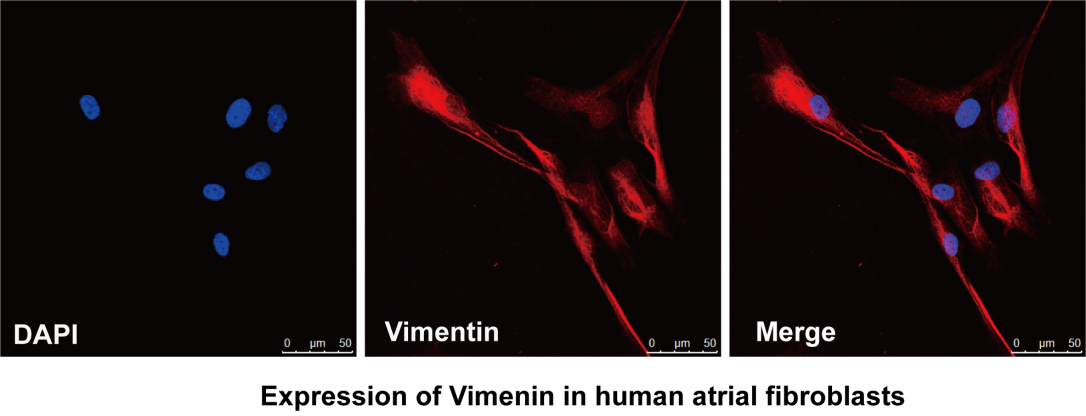
**

**Figure S2: The identification of human atrial fibroblasts.**


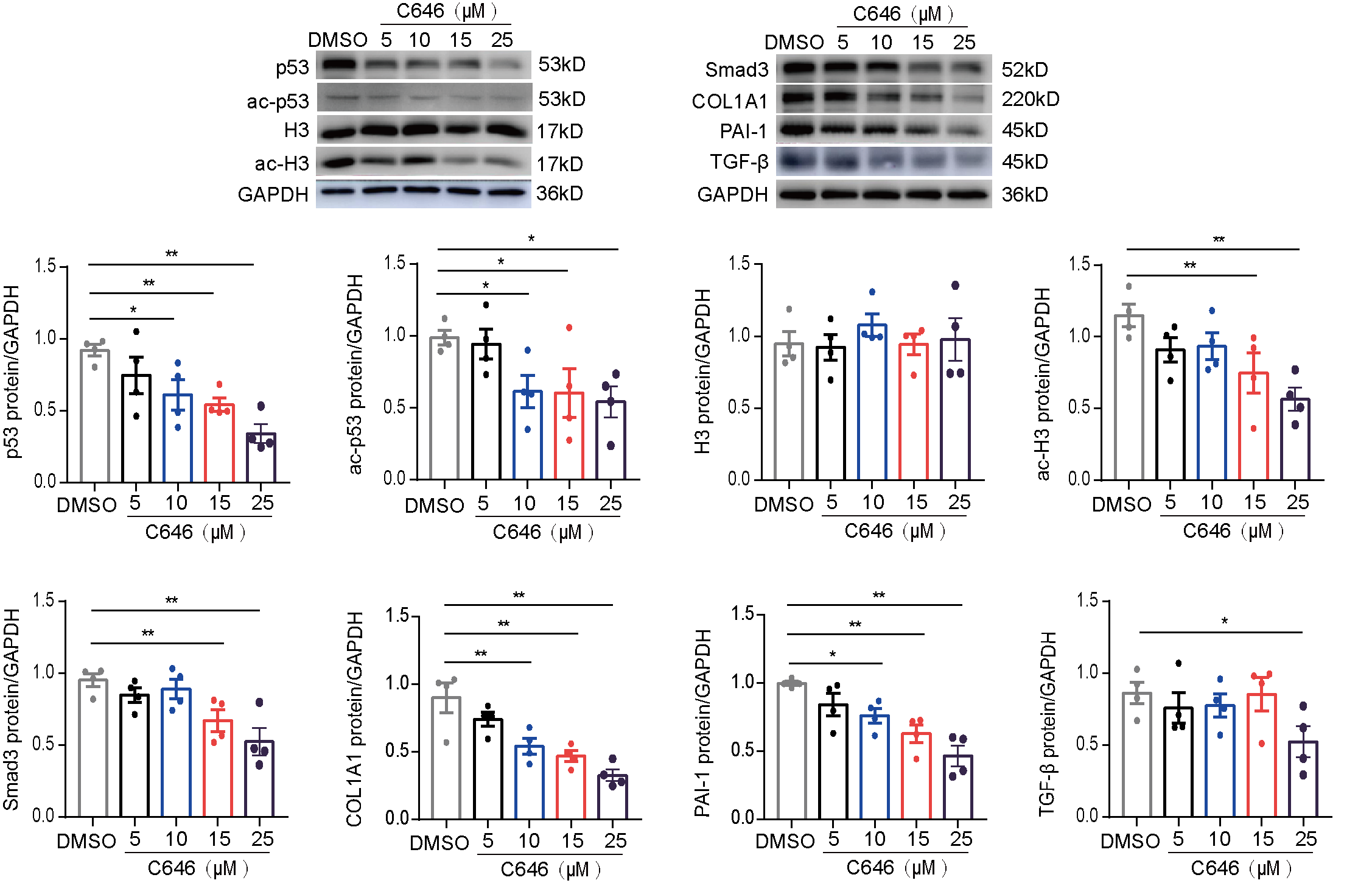


**Figure S3: Selective histone acetyltransferase p300 inhibitor-C646 inhibited the expression of acp53/p53, ac-H3 and fibrosis associated proteins in senescent human atrial fibroblasts.** Representative immunoblots and densitometric analysis of the expression of acp53/p53, ac-H3 and fibrosis associated proteins in senescent human atrial fibroblasts (P11) treated with 5, 10,15 or 25μM C646-treated or control cells. * *P* < 0.05, ** *P* < 0.01; Data are mean ± s.e.m.


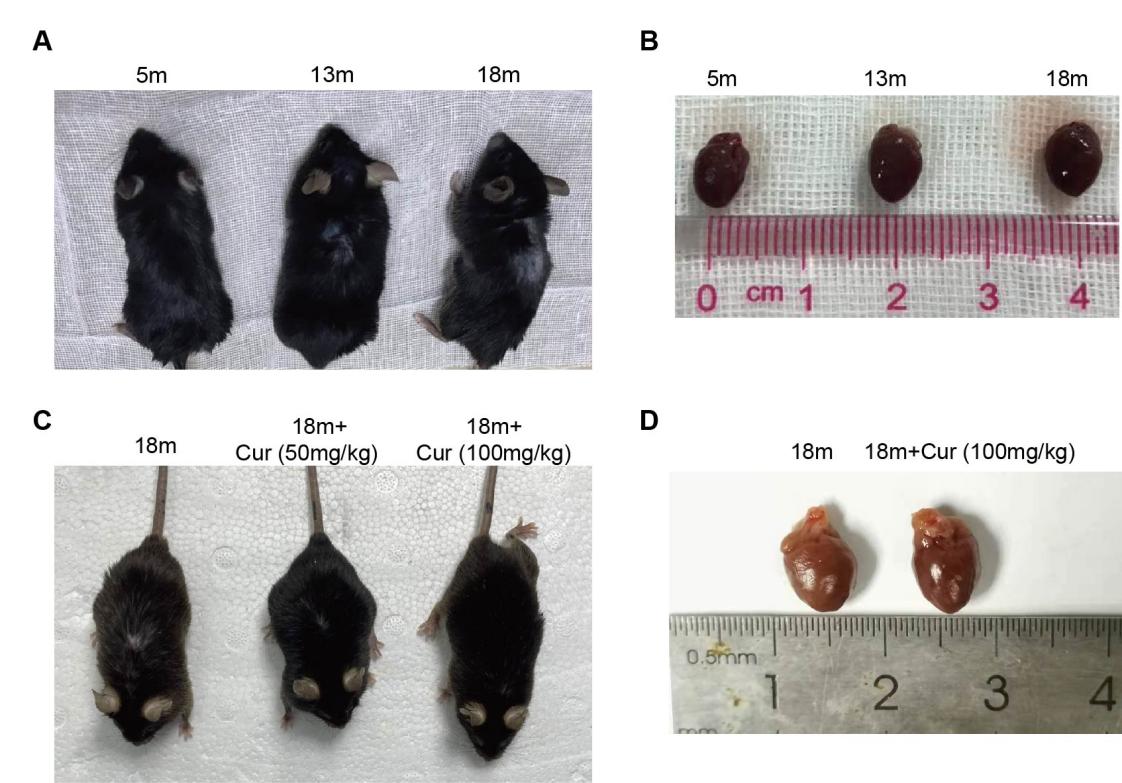


**Figure S4: The body and heart appearance of young and old mice treated with or without curcumin (100mg/kg).**

**
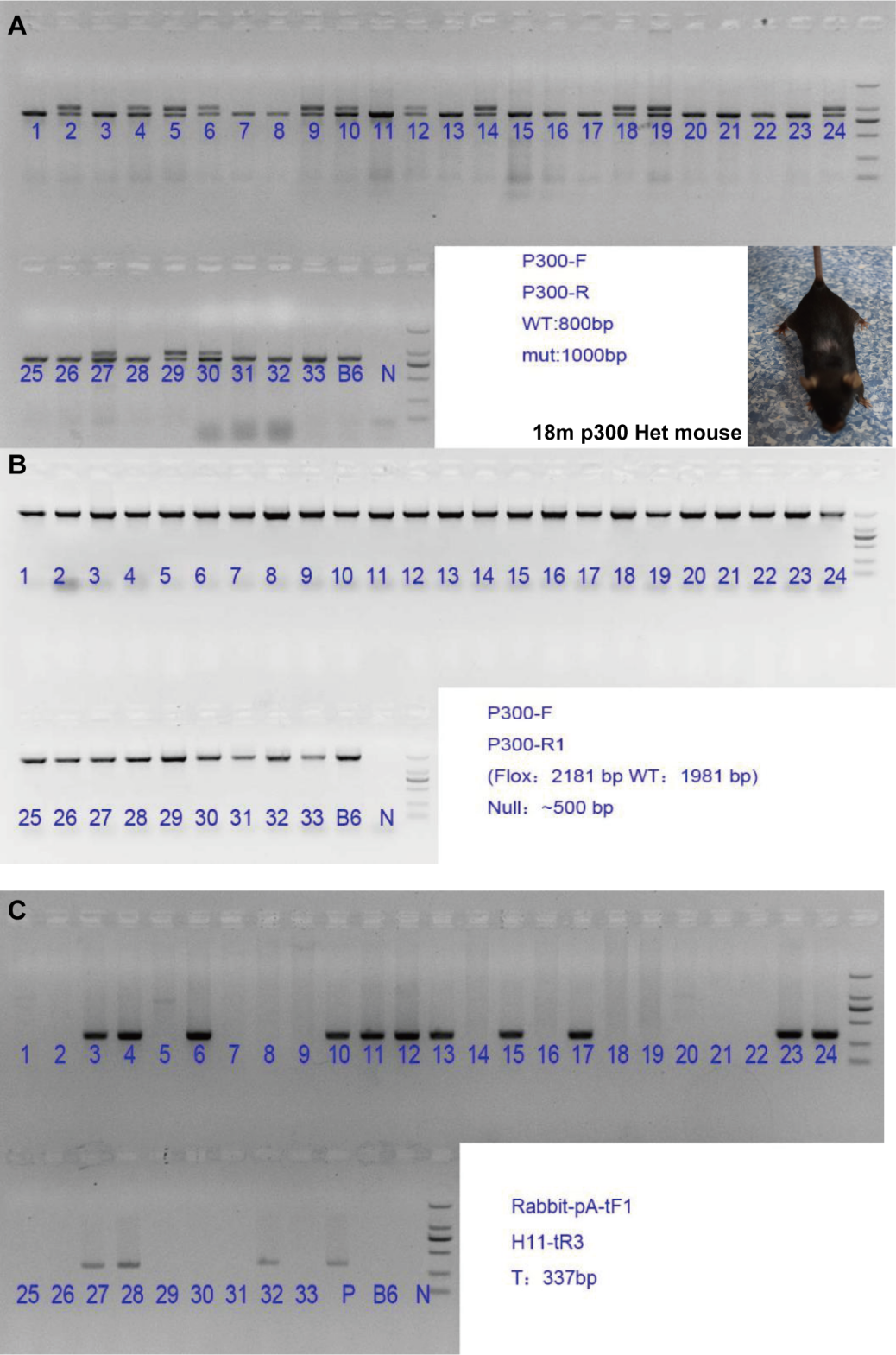
**

**Figure S5: The Identification of mice genotypes.** A. The detection of p300. p300 Mut/Wt：2，4-6，9，10，12，14，18，19，24，27，29，30; Wt/wt：The rest. B. The detection of CAG-cre. T:3，4，6，10-13，15，17，23，24，27，28，32; W: The rest. C. The detection of null.

P: Positive Control; B6：Negative Control，(B6 mouse genome was used); N：No-Template Control

DL2000 Marker：2000bp\1000bp\750bp\500bp\250bp\100bp

**Table S1. Summary of GEO datasets** **information**

| **Catalog** | **GSE115574** | **GSE79768** | **GSE14975** | **GSE41177** |
| --- | --- | --- | --- | --- |
| **Organism** | Homo sapiens | Homo sapiens | Homo sapiens | Homo sapiens |
| **Tissue** | atrial tissue | atrial tissue | atrial tissue | atrial appendage |
| **Experiment type** | Array | Array | Array | Array |
| **Platforms** | GPL570 | GPL570 | GPL570 | GPL570 |
| **Sample(number)** |  |  |  |  |
| **SR** | 29 | 13 | 5 | 3 |
| **AF** | 30 | 13 | 5 | 16 |
| **Total** | 59 | 26 | 10 | 19 |

SR: Sinus rhythm; AF: Atrial fibrillation.

**Table S2.** **Anatomic, Electrophysiology** **and Functional Data (18m C57BL/6 mice treated with curcumin)**

|  | **18m** | **18m+Cur.(50mg/kg/d)** | **18m+Cur.(100mg/kg/d)** |
| --- | --- | --- | --- |
| **n** | 10 | 10 | 10 |
| **Body weight (g）** | 32.19 ± 0.90 | 32.51 ± 1.33 | 35.45 ± 1.29 |
| **Heart wt/TL (mg/mm)** | 9.34 ± 0.61 | 8.83 ± 0.33 | 8.70 ± 0.13 |
| **HR (bpm)** | 586.55 ± 17.32 | 550.77 ± 38.27 | 541.45 ± 17.10 |
| **PWD (ms)** | 20.00 ± 1.12 | 19.90 ± 1.19 | 20.20 ± 0.81 |
| **PR interval (ms)** | 50.70 ± 2.00 | 46.10 ± 1.58 | 45.50 ± 1.28 ⃰ |
| **SNRT (ms)** | 193.78 ± 8.79 | 194.30 ± 8.92 | 174.70 ± 7.14 |
| **CSNRT (ms)** | 57.68 ± 3.59 | 52.37 ± 5.96 | 42.59 ± 5.01 ⃰ |
| **Total AF duration (s)** | 272.12 | 78.17 | 4.217 |
| **n** | 19 | 12 | 17 |
| **LVEDD (mm)** | 2.67 ± 0.13 | 2.74 ± 0.19 | 2.52 ± 0.13 |
| **LVESD (mm)** | 3.55 ± 0.12 | 3.74 ± 0.17 | 3.56 ± 0.12 |
| **LVd (μL)** | 54.67 ± 4.74 | 61.93 ± 5.85 | 54.52 ± 4.32 |
| **LVs (μL)** | 28.25 ± 3.47 | 30.51 ± 4.25 | 24.54 ± 3.22 |
| **LVEF (%)** | 50.77 ± 2.21 | 53.91 ± 3.72 | 57.52 ± 2.50 |
| **LVFS (%)** | 25.50 ± 1.35 | 27.96 ± 2.54 | 29.94 ± 1.61 |

HR, heart rate; PWD, p wave duration; AERP, atrial effective refractive period; SNRT,sinus node recovery time; CSNRT, corrected SNRT; LVEDD: left ventricular end-systolic dimension；LVESD: left ventricular end-diastolic dimension; LVd: left ventricular diastolic volume; LVs: left ventricular systolic volume; LVEF: left ventricular ejection fraction; LVFS: left ventricular fractional shortening;

Data are mean±SEM, * *p* < 0.05 *vs.* 18m.

**Table S3. Anatomic, Electrophysiology** **and Functional Data (WT and p300 KO Het mice)**

| **Genotype** | **7mW** | **7mT** | **18mW** | **18mT** |
| --- | --- | --- | --- | --- |
| **n** | 10 | 10 | 10 | 10 |
| **Body weight (g）** | 29.14 ± 1.28 | 27.39 ± 1.30 | 46.04 ± 3.59** | 38.16 ± 2.58 ⃰ |
| **Heart wt/TL (mg/mm)** | 8.34 ± 0.49 | 8.71 ± 0.64 | 9.40 ± 0.42 | 8.94 ± 0.48 |
| **HR (bpm)** | 569.02 ± 10.15 | 549.51 ± 21.50 | 601.60 ± 23.04 | 549.23 ± 26.84 |
| **PWD (ms）** | 19.40 ± 0.67 | 18.20 ± 0.81 | 23.20 ± 0.63** | 19.70 ± 0.45＃＃ |
| **PR interval (ms)** | 40.90 ± 0.87 | 42.7 ± 1.13 | 53.50 ± 2.37** | 47.60 ± 2.11*＃ |
| **SNRT (ms)** | 216.90 ± 12.15 | 209.90 ± 10.47 | 201.70 ± 17.63 | 218.00 ± 12.38 |
| **CSNRT (ms)** | 40.88 ± 5.47 | 43.27 ± 3.22 | 66.10 ± 8.97** | 64.04 ± 5.60* |
| **Total AF duration(s)** | 1.47 | 2.16 | 75.11 | 6.31 |
| **LVEDD (mm)** | 3.30 ± 0.13 | 3.34 ± 0.11 | 3.52 ± 0.15 | 3.39 ± 0.16 |
| **LVESD (mm)** | 2.18 ± 0.11 | 2.24 ± 0.10 | 2.46 ± 0.15 | 2.33 ± 0.18 |
| **LVd (μl)** | 45.10 ± 4.48 | 46.20 ± 3.45 | 53.07 ± 5.66 | 48.73 ± 5.90 |
| **LVs (μl)** | 16.52 ± 2.26 | 17.48 ± 1.90 | 22.86 ± 3.76 | 20.63 ± 4.23 |
| **LVEF (%)** | 64.40 ± 1.61 | 62.85 ± 1.99 | 58.30 ± 2.76 | 60.52 ± 3.71 |
| **LVFS (%)** | 34.30 ± 1.11 | 33.24 ± 1.35 | 30.39 ± 1.94 | 31.99 ± 2.46 |

Data are mean±SEM; * *p* < 0.05, ** *p* < 0.01 *vs.* 7mW, ^#^ *p* < 0.05, ^#^ ^#^ *p* < 0.05 *vs.* 18mW .
